# Supplementary material for: Mechanochemical feedback control of dynamin independent endocytosis modulates membrane tension in adherent cells
Source: Nat Commun. 2018 Oct 11;9:4217. doi: 10.1038/s41467-018-06738-5 (PMC6181995; doi:10.1038/s41467-018-06738-5)
Supplement: Supplementary file 1 — Supplementary Information [file 41467_2018_6738_MOESM1_ESM.pdf]

Mechanochemical feedback control of dynamin independent endocytosis modulates membrane tension in adherent cells

Thottacherry et al.

SUPPLEMENTARY INFORMATION

SUPPLEMENTARY NOTE 1:

MECHANOCHEMICAL MODEL FOR THE CONTROL OF CELL MEMBRANE TENSION

The mechanochemical model involves (i) Membrane flux via CG endocytosis, (ii) concentration of vinculin in the open state, and (iii) concentration of activated GBF1, which together go to sense and regulate the membrane tension.

To start with we propose the following constitutive relation between the membrane tension γ and membrane area A

$$\gamma = \gamma_0 - k_s(A - A_0(V, A_c, \dots)) \tag{1}$$

Where k_s is the area stretch modulus and A_0 depends on other variables fixed by the environment, such as the volume V (or osmotic pressure-π), the extent of area A_c adhered to the substrate, and so on. We assume that these quantities do not change during the dynamics of A. The above constitutive equation is valid when the cell membrane is very floppy, i.e., for instance when $A \gg A_0(V)$ (where $A_0 \sim V^{2/3}$), thus since γ_0 is the tension when $A = A_0$, this implies $\gamma \ll \gamma_0$.

Vinculin exists in an *open* or *closed* configuration[1,2], with number fractions V_o, V_c, respectively. The transition rates between these states depend on the force applied on the Integrins[3]; since this force goes to change the membrane tension (amongst other effects), we will assume that the transition rates between these states depends on the instantaneous tension of the cell membrane. However, it should be understood that in the absence of the integrin force-transduction machinery, vinculin would not be able to respond to membrane tension[4] (and Fig. 7d, 7e of manuscript). With this understanding, we propose the following dynamics for the vinculin concentration,

$$\frac{dV_o}{dt} = k_{c\to o}(\gamma)(1 - V_o) - k_{o\to c}V_o \tag{2}$$

where we have fixed the total vinculin levels and set $V_o + V_c = 1$. The activation rate $k_{c\to o}(\gamma) = k_v\gamma(K_v + \gamma)$ is assumed to depend on tension [1] that operates over a dynamical range set by K_v . If we take this dynamics to be fast[5,6] , then the levels of vinculin in the open configuration is an indicator of the instantaneous tension of the cell membrane,

$$V_o = \frac{k_{c\to o}(\gamma)}{k_{c\to o}(\gamma) + k_{o\to c}} \tag{3}$$

Vinculin is thus a *sensor* of the instantaneous membrane tension. This is consistent with the reduction in endocytic rate upon use of the constitutively active vinculin (Fig 7e). Further, our finding that the endocytic rates do not change upon lowering of membrane tension in this case suggest that the converse holds, namely that the absence of vinculin (or expression of the constitutively inactive) significantly affects the cell's ability to respond to changes in tension (Fig 7a and 7d).

Activated GBF1 is a GEF for ARF1 which drives membrane area uptake via CG endocytosis[7]. Our experiments suggest that the activated GBF1 is associated with formation of punctae of GBF1 and that increasing the levels of V_o inhibits the number of these punctae (Fig 6a, 6b and 7f). This would imply that increasing the levels of V_o inhibits the activation of GBF1, leading to the following dynamics for the concentration of activated GBF1, G_{act} ,

$$\frac{dG_{act}}{dt} = k_{act}(V_o)(1 - G_{act}) - k_{in}G_{act} \quad (4)$$

where $k_{act}(V_o) = k_g/(K_g + V_o)$, which decreases with increasing V_o. This is consistent with the observation that vinculin null cells have higher levels of activated GBF1 (Supplementary Fig 11c).

Finally, the dynamics of membrane tension is set by the dynamics of internalisation of membrane area via Eq. 1, from which it follows that $dA/dt \propto -d\gamma/dt$. The flux balance of cell membrane area is then equivalent to,

$$\frac{d\gamma}{dt} = k_{end}(\gamma, G_{act}) - k_{exo}(\gamma) \quad (5)$$

where the endocytic rate depends on the levels of activated GBF1 and the membrane tension - it increases with increasing GBF1 and decreases with increasing tension,

$$k_{end}(\gamma, G_{act}) \propto \frac{G_{act}}{(K_m+\gamma)(K_c+G_{act})} \quad (6)$$

while the exocytic rate $k_{exo}(\gamma)$ is an increasing function of γ [5], presumably regulated via a signalling mechanism.

Note, in the above we have taken the observation (Fig. 7f) to imply that GBF1 levels do not *directly* depend on tension; its dependence is via V_o. This suggests a separation of time scales between the dynamics of vinculin and GBF1 (and hence membrane tension). Further, the tacit assumption in our analysis is that variations in V_o and G_{act} do not change A_0 (Eq. 1).

The above description is consistent with a simple feedback inhibition (Fig. 8b). This negative feedback between the state of a membrane and the protein comprises a hybrid of two types of interactions - mechanical and chemical - one being slow (activation), the other being fast (inhibition).

To understand how such a feedback inhibition can provide a robust feedback control, we need to be able to track the set point tension. The tension set point γ_s should depend on the parameters of the machine model and its interaction with that part of the environment which is not changing over the time scale of the processes under study. Thus it should depend on the parameters that describe the dynamics of the cell and the parameters in A_0. To be able to provide a feedback control, the controller must compare the instantaneous value of the tension γ with the set point tension γ_s and compensate for this difference. For the feedback control shown in Fig. 8b of the main text, there are two possibilities discussed below:

1. Vinculin senses γ via its concentration V_o (Eq. 3) and the error $V_o - V_s$, where

$$V_s \equiv V_o(\gamma_s) = \frac{k_{c\to o}(\gamma_s)}{k_{c\to o}(\gamma_s)+k_{o\to c}} \quad (7)$$

is fed to the activated GBF1 via $k_{act}(V_o) = k_{act}/(K_G + (V_o - V_s))$. This implies that when $V_o(\gamma) - V_s > 0$ $(V_o(\gamma) - V_s < 0)$, there is a decrease (increase) in GBF1 and a concomitant decrease (increase) in endocytosis, keeping the exocytosis constant – this will lead to a decrease (increase) in γ and hence a decrease (increase) in $V_o(\gamma)$. This feedback control is chemical.

2. The feedback control could be mechanical and operate between the GBF1 and the CG endocytosis nodes. Thus,

$$\frac{d\gamma}{dt} = k_{end}(\gamma, G_{act}) - k_{exo}(\gamma) \quad (8)$$

where the endocytic rate depends on the levels of activated GBF1 and the membrane tension - it increases with increasing GBF1 and decreases with increasing tension,

$$k_{end}(\gamma, G_{act}) \propto \frac{G_{act}}{(K_n+(\gamma-\gamma_s))(K_g+G_{act})} \quad (9)$$

while the exocytic rate can be taken to be tension dependent.

While many engineering applications rely on an asymptotic control analysis (where the set point is reached as $t \to \infty$), in the cellular context, any control mechanism should be able to track the set point in finite time, giving rise to the notion of optimal control. A detailed mathematical analysis of such optimal control is outside the scope of this paper and will be presented elsewhere.

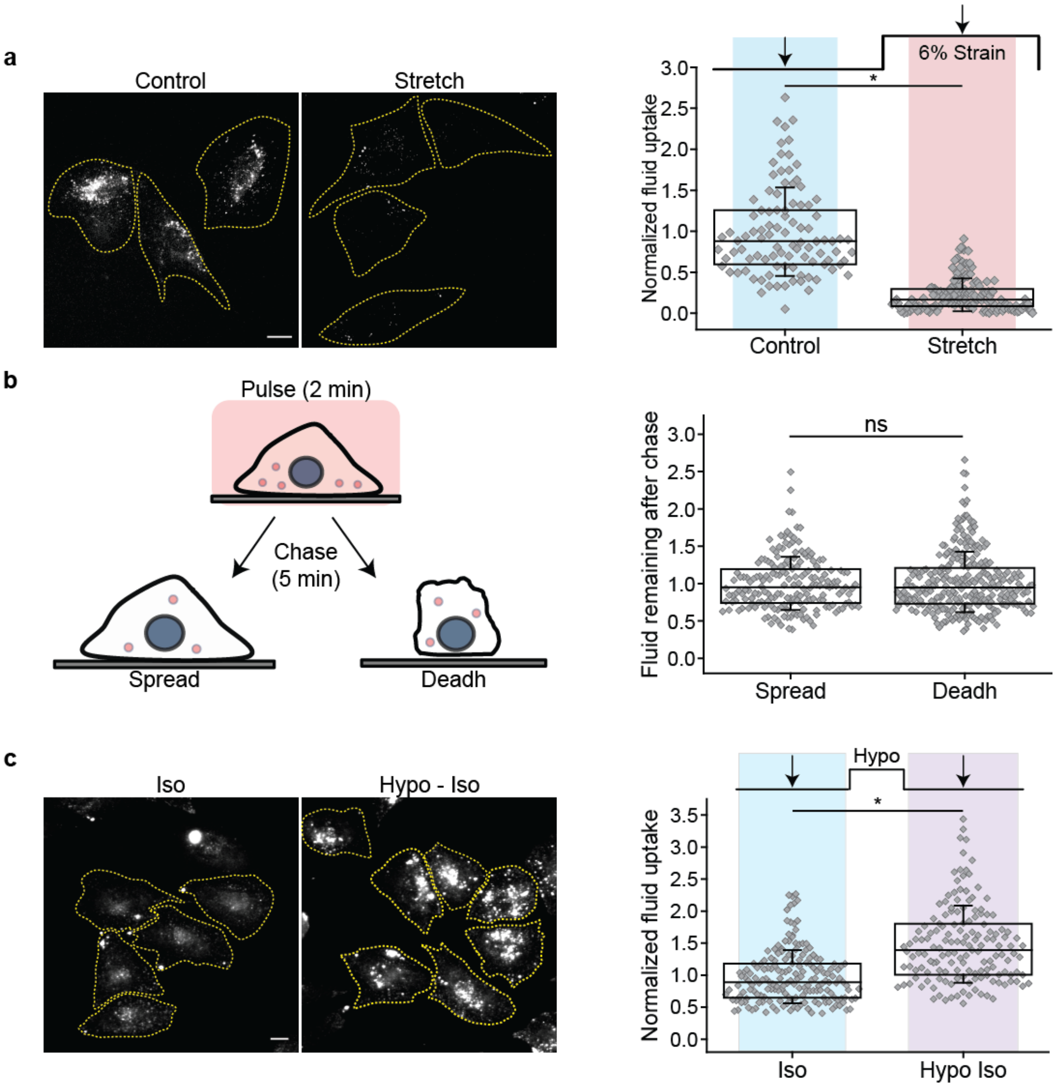

Supplementary Figure 1: Endocytosis on tension modulation (a) Fluid uptake in WT – MEF subjected to 6% strain for 90 seconds. Fluid uptake is normalized to the cells that were not subjected to the strain (control). (n = Control (97), Stretch (167)). **(b)** Cartoon (left) describes the pulse chase protocol used to look at recycling during deadhering. CHO cells are pulsed with TMR-Dex for 2 minutes, washed and chased for 5 minutes either in their adhered steady state (Spread) or during de-adhering (Deadh) were washed, fixed, and imaged. Box plot (right) shows TMR-Dex remaining in the cells after chase in the deadhering or in spread condition normalized to the spread condition. (n = Spread (185), Deadh (280)). **(c)** Fluid uptake for 1 minute either in steady state (Iso) or after hypotonic shock (hypo-iso) for one minute. Images (left) of endocytosed TMR-Dex and box plot (right) show the extent of fluid-phase uptake. (n = Iso (178), Hypo Iso (151)). Box plot shows median, 25th and 75th percentile, and whiskers show the standard deviation. Individual data points are overlaid on box plot where each data point is the mean intensity per cell. 'n' indicates total number of cells in each condition pooled from two different experiments with duplicates per experiment. *: $P < 0.001$, ns: not significant by Mann-Whitney U test. Scale bar, 10 µm.

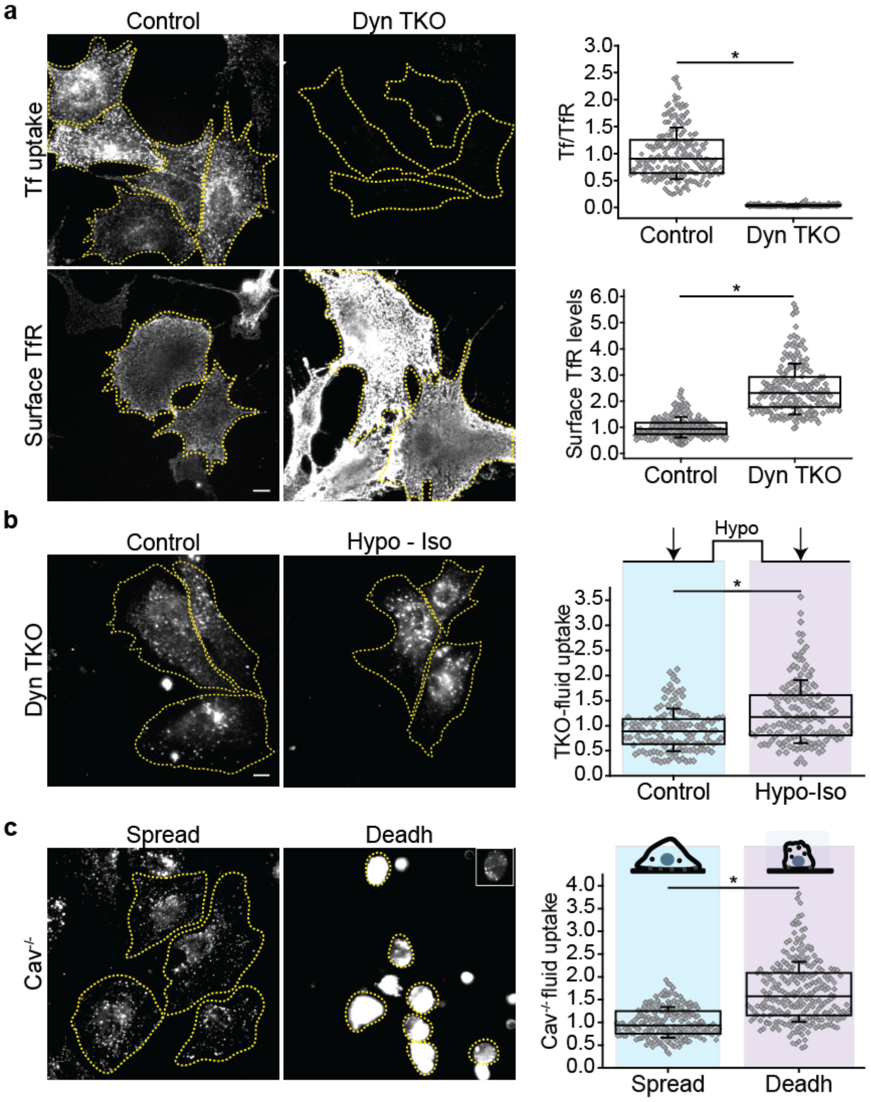

Supplementary Figure 2: Endocytic response in Dynamin and caveolin null cells (a) Dynamin TKO cells, or Control was pulsed with Tf-A568 for 5minute, surface stripped, labelled with antibody against transferrin receptor (TfR), washed and fixed. Box plot (right) shows transferrin (Tf) uptake normalized to the TfR surface levels (top) and surface TfR levels normalized to the control (bottom). Corresponding wide field images (left) show extent of endocytosed TfR (top) and surface TfR (bottom). Removal of all Dynamin isoforms inhibits transferrin uptake comprehensively while increasing surface levels of TfR. (n = Control (235), Dyn TKO (203)). **(b)** Fluid uptake in dynamin TKO cells for 1 minute either in steady state (control) or after hypotonic shock (hypo-iso). Wide-field images (left) show extent of TMR-Dex uptake and the box plot (right) shows fluid-phase uptake per cell in hypo-iso condition normalized to the control. Note while uptake of TfR is completely inhibited (a), fluid-phase uptake (b) exhibits a typical increase on shifting from hypotonic to isotonic conditions in Dynamin TKO cells. (n = Control (141), Hypo Iso (158)). **(c)** Fluid uptake in caveolin null MEFs (Cav-/-) either in adherent (Spread) or during detachment (Deadh). (n = Spread (223), Deadh (254)). Box plot shows median, 25th and 75th percentile, and whiskers show the standard deviation. Individual data points are overlaid on box plot where each data point is the mean intensity per cell. 'n' indicates total number of cells in each condition pooled from two different experiments with duplicates per experiment. *: $P < 0.001$, ns: not significant by Mann-Whitney U test. Scale bar, 10 μm (a, b, c).

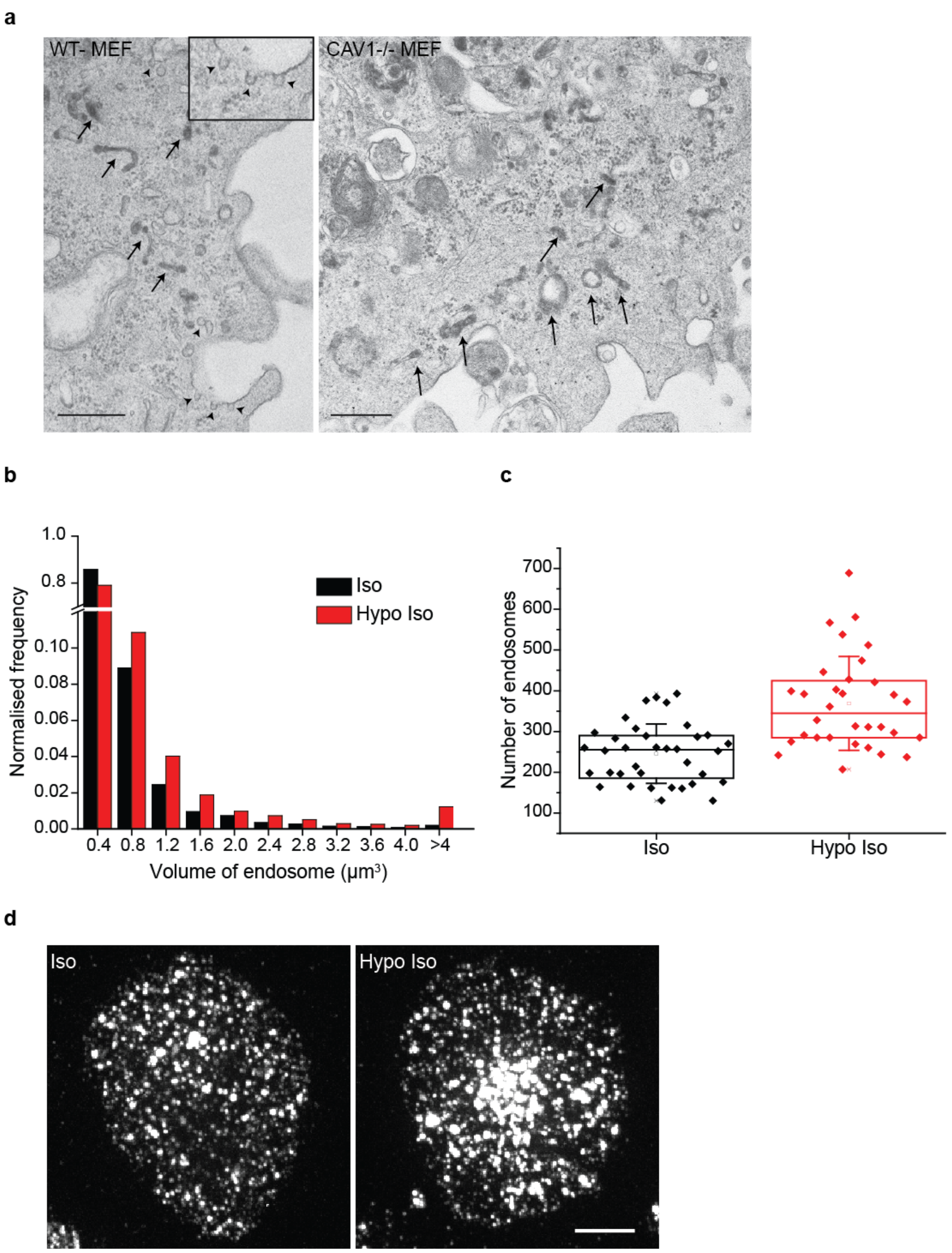

Supplementary Figure 3: Characterization of endosomes on reducing tension (a) Electron micrographs of WT-MEF and Cav1-/- MEF on deadhering show similar CG endosomes. CTxB-HRP uptake for 5 minutes and processed for DAB reaction as described in methods is done in both wild type MEF (WT-MEF) and Cav1-/- MEF. Arrows show internalized CG carriers in both cell types and arrow head show surface connected caveolae in de-adhered WT cells (inset shows zoomed in image). Scale bar, 500nm. **(b)** The cells were pulsed with TMR-Dex for one minute either in Iso after a hypotonic shock of 1minute and compared to cells pulsed in Iso for 1 minute. The fraction of endosomes based on size of the endosome is plotted as histogram (Black – Iso, Red – Hypo Iso). **(c)** Number of endosomes per cell is plotted as box plot from (b) and whiskers indicate standard deviation (Iso- 35 cells, Hypo Iso - 32 cells). **(d)** Maximum intensity projection of confocal stacks (see Supplementary Movie 1 and 2) show representative image of endosomes in either Iso or Hypo Iso as quantified in (b) and (c). Scale bar, 5 μm.

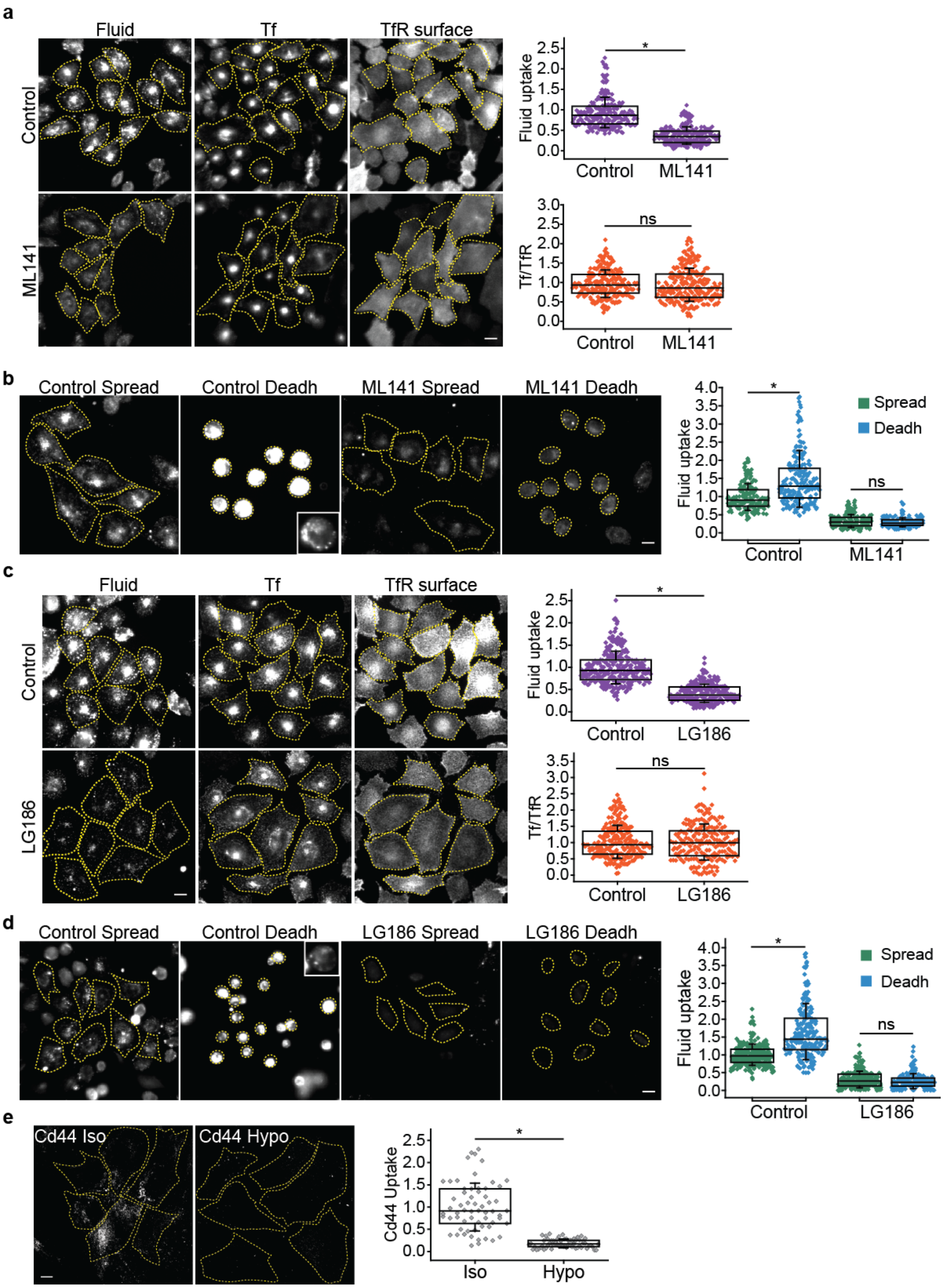

Supplementary Figure 4: Role of CG pathway in tension response (a) Fluid and Transferrin uptake in CHO cells treated with DMSO (Control) or with ML141 (10µM) for 45 minutes. Cells were pulsed for 5 minutes with TMR-Dex (Fluid) and A488-Tf (Tf), washed and surface-stripped of remnant-cell surface Tf. These cells were incubated with A647-

labelled OKT9 antibody on ice to detect surface transferrin receptor (TfR Surface). (n = Fluid uptake – Control (219), ML141 (258); Tf/TfR – Control (215), ML141 (239)). **(b)** Adherent CHO cells treated without (Control) or with ML141 (10μM) for 45 minutes were pulsed for 5 minutes with TMR-Dex without detachment (Spread) or during the deadhering process (Deadh), and washed extensively, fixed and taken for imaging. Images (left) of endocytosed TMR-Dex and box plot (right) show the extent of fluid-phase uptake normalized to that observed in the control spread cells. Note that increase in endocytosis observed while deadhering is completely abolished upon inhibition of CG endocytosis by ML141. (n = Control- Spread (183), Deadh (225); ML141- Spread (219), Deadh (214)). **(c)** CHO cells treated without (Control) or with LG186 (10μM) for 30 minutes were pulsed for 5 minutes with TMR-Dex (Fluid) and A488-Tf (Tf), washed extensively and surface-stripped of remnant-cell surface Tf. These cells were incubated with A647-labelled OKT9 antibody to detect surface transferrin receptor (TfR Surface). Wide field images of cells (left) and box plot of total fluid-phase uptake and Tf uptake normalized to surface receptor level shows that the effect of LG186 was only on the fluid-phase uptake but not on TfR endocytosis. (n = Fluid uptake- Control (283), LG186 (274); Tf/TfR- Control (225), LG186 (194)). **(d)** Adherent CHO cells treated without (Control) or with LG186 (10μM) for 30 minutes were pulsed for 5 minutes with TMR-Dex without detachment (Spread) or during the deadhering process (Deadh), and washed, fixed and taken for imaging. Images (left) of endocytosed TMR-Dex and box plot show the extent of fluid-phase normalised to that observed in the control cells. Note that increase in endocytosis observed while deadhering is completely abolished upon inhibition of CG endocytosis by inhibiting GBF1 using LG186. (n = Control- Spread (261), Deadh (224); LG186- Spread (276), Deadh (178)). **(e)** WT MEFs were pulsed CD44 mAb for 2 minutes either in isotonic medium (Iso) or in 75mOsm hypo-osmotic medium (Hypo) at 37°C, acid washed to strip surface mAb, washed and labeled with AF-555 secondary antibody. Box plot (right) shows per cell uptake in hypo-osmotic situation normalized to the isotonic situation for the representative images shown on left. (n = Iso (62), Hypo (62)). Box plot shows median, 25th and 75th percentile, and whiskers show the standard deviation. Individual data points are overlaid on box plot where each data point is the mean intensity per cell. 'n' indicates total number of cells in each condition pooled from two (a, b, c, d) or three (e) different experiments with duplicates per experiment. *: $P < 0.001$, ns: not significant by Mann-Whitney U test. Scale bar, 10 μm.

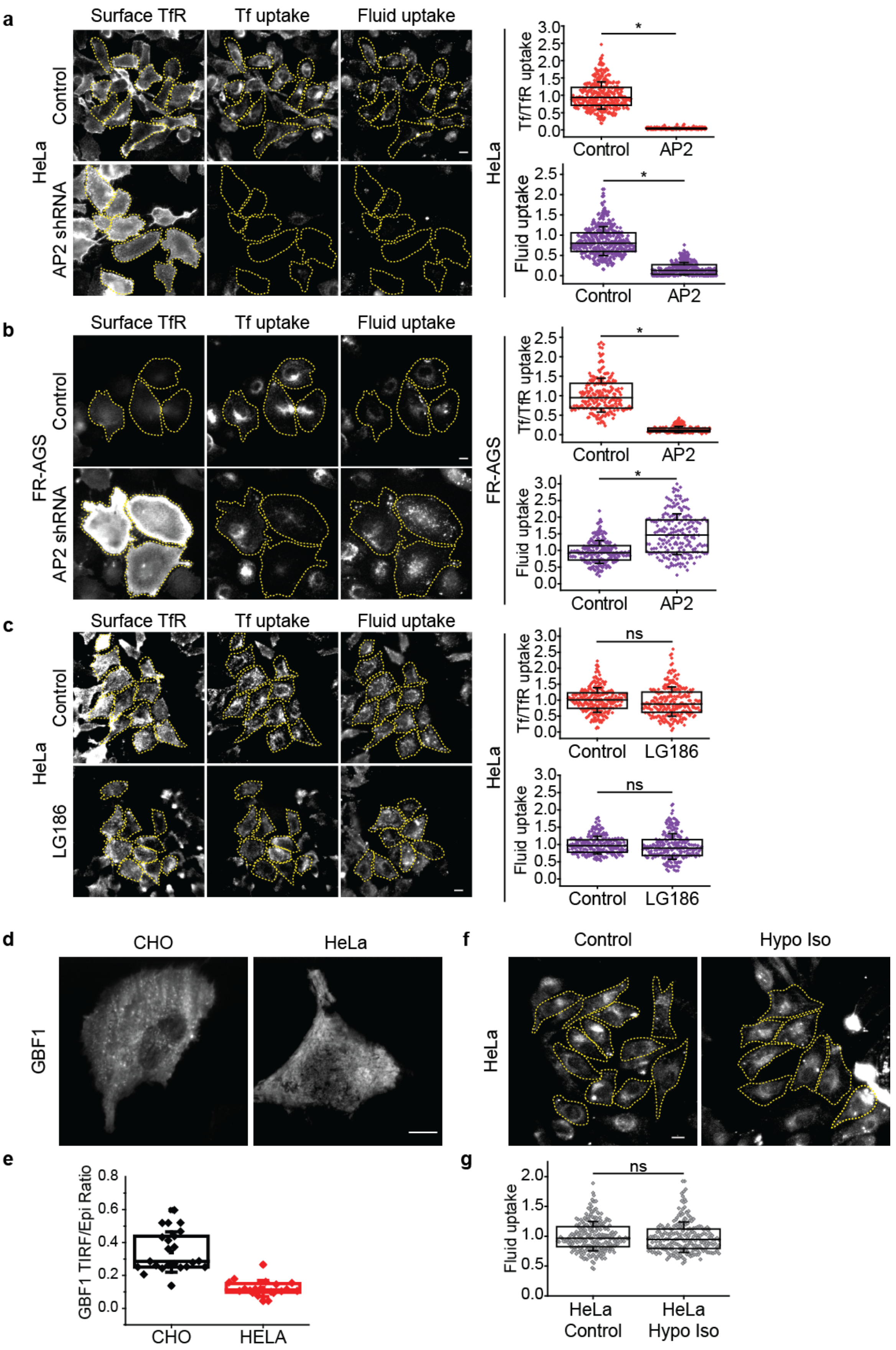

Supplementary Figure 5: HeLa cells lack a bonafide CG pathway (a) HeLa cells transfected with AP2 shRNA (AP2) or with pSUPER vector (control) for 4 days were pulsed

for 5 minutes with TMR-Dex (Fluid) and A488-Tf (Tf), washed extensively and surface-stripped of remnant-cell surface Tf, followed by labeling with A647-labelled OKT9 antibody to detect surface transferrin receptor (Surface TfR). Left panel shows representative wide field images of cells and right panel shows box plot of total fluid-phase uptake and Tf uptake normalized to surface receptor level. (n = Tf/TfR- Control (376), AP2 (342); Fluid uptake- Control (380), AP2 (342)). **(b)** FR-AGS cells transfected with AP2 shRNA (AP2) or with vector control (control) for 4 days were pulsed for 5 minutes with TMR-Dex (Fluid) and A488-Tf (Tf), washed extensively and surface-stripped of remnant-cell surface Tf, followed by labeling with A647-labelled OKT9 antibody to detect surface transferrin receptor (Surface TfR). Left panel shows wide field images of cells and right panel shows box plot of total fluid-phase uptake and Tf uptake normalized to surface receptor level. (n = Tf/TfR- Control (223), AP2 (203); Fluid uptake- Control (226), AP2 (207)). **(c)** HeLa cells treated with DMSO (Control) or with LG186 (10μM) for 30 minutes were pulsed for 5 minutes with TMR-Dex (Fluid) and washed extensively, or pulsed with A488-Tf (Tf) and surface-stripped of remnant-cell surface Tf, followed by labeling with A647-labelled OKT9 antibody to detect surface transferrin receptor (TfR Surface). Wide field images of cells (left) and box plot (right) of total fluid-phase uptake and Tf uptake normalized to surface receptor level shows that LG186 does not have an effect on the fluid-phase uptake and TfR endocytosis in HeLa cells. (n = Tf/TfR- Control (254), LG186 (245); Fluid uptake- Control (250), LG186 (245)). **(d)** GBF1-GFP was transfected in either CHO or HeLa cells and imaged in TIRF and in wide field (epi-fluorescence) format. HeLa cells do not show surface punctae formation unlike CHO cells in TIRF. **(e)** Quantification of the TIRF to wide field ratio of GBF1-GFP intensity. (n = 23 (CHO) and 20 (HeLa)). **(f/g)** Rapid shifting from hypotonic to isotonic state does not result in an increase in fluid-phase uptake in HeLa Cells. HeLa cells were pulsed with TMR-Dextran (fluid) for 1 minute either in steady state (Iso) or after hypotonic shock of one minute (Hypo-Iso). Images (f) of endocytosed TMR-Dex and box plot (g) show the extent of fluid-phase uptake. (n = HeLa Control (237), HeLa Hypo Iso (223)). Box plot shows median, 25th and 75th percentile, and whiskers show the standard deviation. Individual data points are overlaid on box plot where each data point is the mean intensity per cell. 'n' indicates total number of cells in each condition pooled from two different experiments with duplicates per experiment. *: $P < 0.001$, ns: not significant by Mann-Whitney U test. Scale bar, 10 μm.

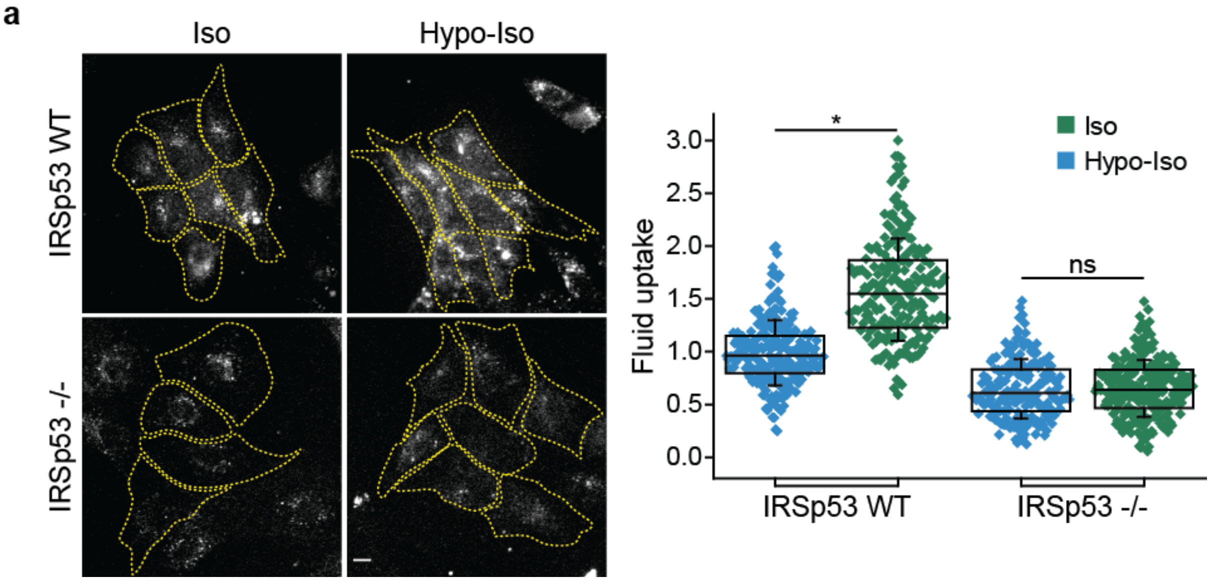

Supplementary Figure 6: IRSp53 is important for the tension response of CG pathway **(a)** Fluid uptake in IRSp53 null MEFs and IRSp53 null MEFs stably expressing IRSP53 WT following a hypotonic shock compared to uptake in isotonic medium (control). Left panel shows representative images and box plot (right panel) shows fluid uptake (for one minute) in IRSp53 -/- (null) and IRSp53 WT (control) compared to uptake after 1minute hypotonic shock (Hypo Iso). (n = IRSP53 WT – Iso (229), Hypo-Iso (209); IRSp53-/- - Iso (199), Hypo-Iso (252)). Box plot shows median, 25th and 75th percentile, and whiskers show the standard deviation. Individual data points are overlaid on box plot where each data point is the mean intensity per cell. 'n' indicates total number of cells in each condition pooled from two different experiments with duplicates per experiment. *: $P < 0.001$, ns: not significant by Mann-Whitney U test. Scale bar, 10 µm.

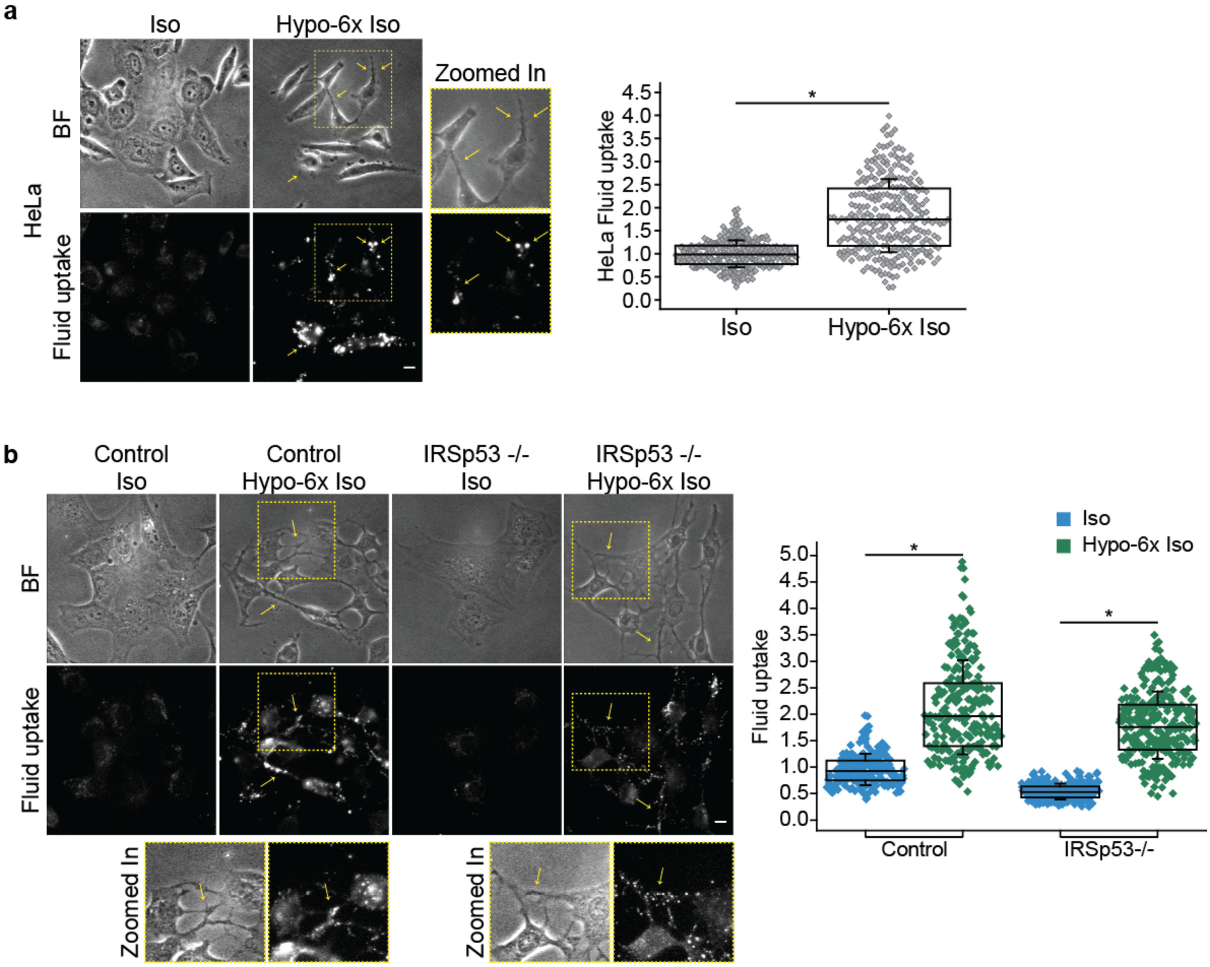

Supplementary Figure 7: CG pathway-independent endocytic response on extreme osmotic shock (a) Fluid uptake in HeLa cells following 10-minute application of 6x diluted hypotonic medium followed by return to isotonicity (Hypo-6x Iso) compared to uptake for 10minutes in isotonic medium (Iso). Left panel shows images of bright field (BF) and fluid uptake in HeLa cells and right panel shows box plot of the same. Selected cells (yellow box) is zoomed in and shown separately with arrows indicating fluid endosomes outside the cell body. (n = Iso (304), Hypo-6x Iso (284)). **(b)** Fluid uptake in IRSp53 -/- or IRSp53 WT (control) either in isotonic medium for 10minutes (Iso) or following the Hypo-6x Iso protocol (Hypo-6x Iso). Left panel shows representative images of bright field (BF) and fluid uptake while right panel shows box plot of fluid uptake normalized to isotonic control. Selected area (yellow box) is zoomed in and shown separately (bottom panel) with arrows indicating fluid endosomes outside the cell body. (n = Control- (Iso (198), Hypo-6x Iso (241); IRSp53-/- -(Iso (244), Hypo-6x Iso (275)). Box plot shows median, 25th and 75th percentile, and whiskers show the standard deviation. Individual data points are overlaid on box plot where each data point is the mean intensity per cell. ‘n’ indicates total number of cells in each condition pooled from two different experiments with duplicates per experiment. *: $P < 0.001$, ns: not significant by Mann-Whitney U test. Scale bar, 10 µm.

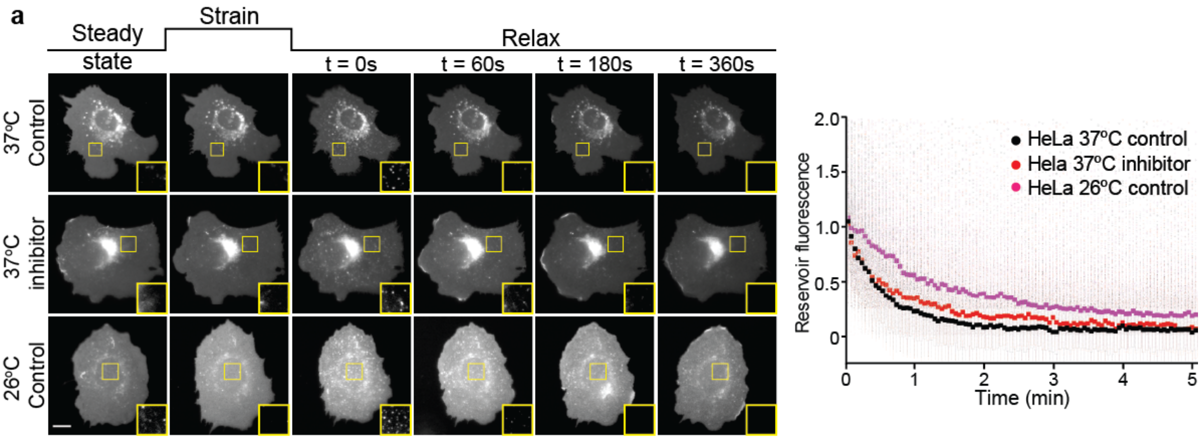

Supplementary Figure 8: Reservoir adsorption in HeLa cells does not respond to GBF1 inhibition. (a) The reservoir fluorescence intensity after stretch relax of HeLa cells transfected with a fluorescent membrane marker (pEYFP-mem) was quantified as a function of time at 37°C in the absence (37°C control) or presence of LG186 (37°C inhibitor), or at room temperature (26°C control). Each point represents median reservoir intensity from more than 200 reservoirs from at least 10 cells. Note while treatment with LG186 had no effect on the rate of reservoir resorption, lowering of the temperature also had only a marginal reduction in the rate of resorption. Scale bar, 10 µm.

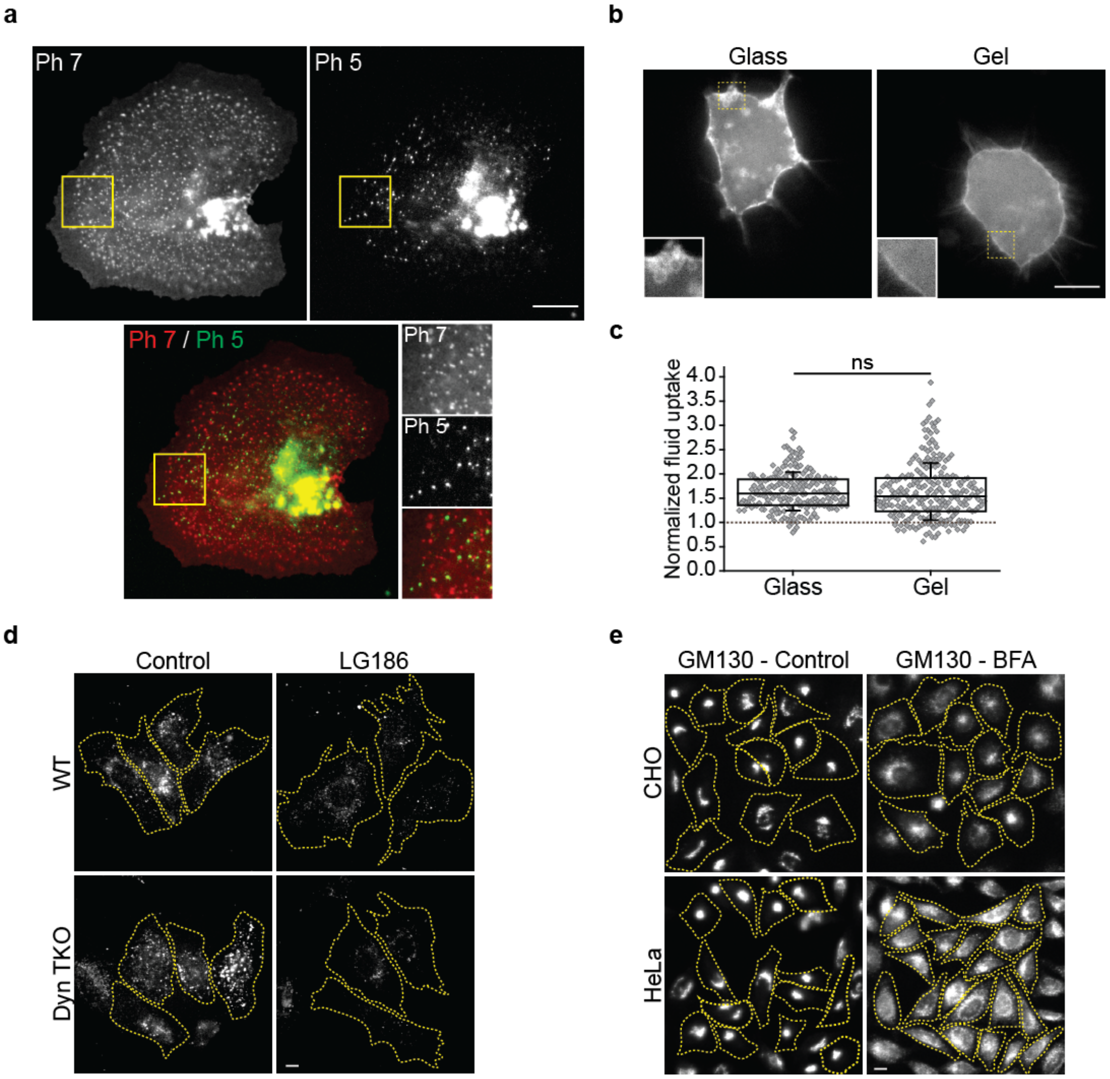

Supplementary Figure 9: Reservoir or VLD formation is not necessary for endocytic response (a) A pH sensitive SecGFP-GPI transfected CHO cells are stretched for one minute and relaxed in pH 7.4 buffer (pH 7, top left) at 37°C showing formation of reservoirs as before. The pH is made acidic (pH 5.5) after 30 seconds to capture newly formed endosomes after stretch-relax (pH 5, top right). The reservoirs do not colocalize with the newly formed endosomes (bottom, insets). **(b)** The cells transfected with CAAX-GFP to mark the membrane are plated on glass (left) or polyacrylamide gels. Cells were incubated with hypo-osmotic medium for one minute followed by isotonic recovery. Images of VLDs are observed only in the cells grown on glass. Insets show a magnification of the edge of the cells where VLDs are expected to form. **(c)** Cells were incubated with TMR-Dextran uptake for 1 minute without (Iso) or with (Hypo-Iso) being subject to hypotonic shock with 1:1 ratio of isotonic medium to water, for one minute and box plot show the extent of fluid-phase uptake. Endocytic pulse of 90 seconds is done either in isotonic situation or after a hypotonic shock in cells plated on glass or gel. Mean uptake per cell after hypotonic shock for either gel or glass is normalized to its uptake in isotonic conditions (dotted line). Data represent the mean intensity per cell from two different experiments with duplicates per experiment. (n = Glass (214), Gel (246)). Box plot shows median, 25th and 75th percentile, and whiskers show the standard deviation. Individual data points are overlaid on box plot where each data point is the mean intensity per cell. 'n' indicates total number of cells in each condition pooled from two different experiments with duplicates per experiment. *: $P < 0.001$, ns: not significant by

Mann-Whitney U test. **(d)** WT or Dynamin TKO cells were treated with LG186 or with the vehicle (DMSO; Control), and incubated with TMR-Dex for 5 minutes, washed and fixed prior to imaging on a Wide Field microscope. The images show that while Dyn TKO cells exhibit a higher fluid-phase uptake that the WT cells, this uptake is sensitive to the inhibition of GBF1, confirming that it is the CG endocytosis that has a higher activity in Dynamin TKO cells (quantified in Fig. 5c). **(e)** BrefeldinA (BFA) treatment causes disruption in GM130 distribution in both HeLa cells and CHO cells. Cells were treated with 20 μg/ml of BFA for 45 minute, fixed, permeabilized and labelled with GM130 antibody to mark cis-Golgi and imaged on a wide field microscope. BFA treatment disrupts the peri-nuclear localization in both CHO and HeLa cells, indicating that BFA is functional in both cell types. Scale bar, 10 μm.

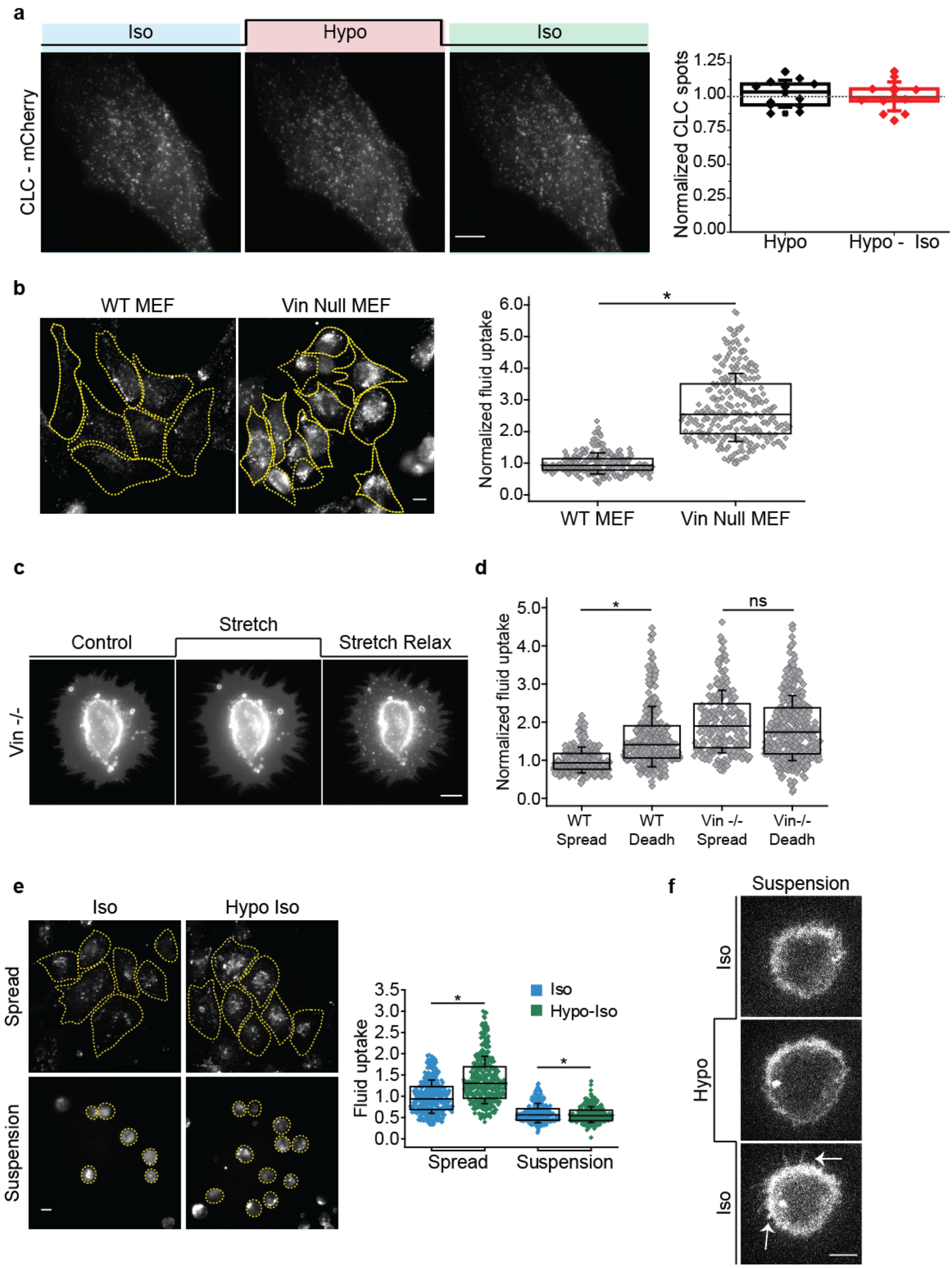

Supplementary Figure 10: Role for vinculin and adhesion in the endocytic tension-response (a) WT cells transfected with CLC-mCherry (Clathrin Light Chain-mCherry) was imaged live using TIRF microscopy during osmotic changes as indicated. Images (Left panel) and graph (right) show the quantification of number of CLC punctae per cell normalized to the steady state number of punctae from 13 cells. Note that the number of clathrin pits at the cell surface do not respond to changes in tension. **(b)** WT or vinculin null (Vin Null) MEFs

are pulsed with F-Dex for 3 minutes as described before at 37°C. Left panel shows quantification of uptake per cell normalized to the WT levels and right panel shows the representative images of the same. In each endocytosis experiment, the data represent the mean intensity per cell from 2 independent experiments with two technical duplicates per experiment. (n = WT (230), Vin Null (256)). **(c)** Vinculin null cells transfected with a fluorescent membrane marker (pEYFP-mem) and plated on PDMS was stretched and relaxed as described in the methods. Stretch – relax shows formation of reservoirs similar to WT cells. **(d)** WT or vinculin null (Vin -/-) MEFs were pulsed with F-Dex either during deadhering or in spread state for 3 minutes and the pulse is stopped using ice cold buffer as described before. De-adhered cells are added to vial containing ice cold buffer and allowed to attach on a fresh coverslip bottom dish to be fixed and imaged. Endosomal intensity per cell is quantified and normalized to the intensity of the WT spread. Note that vinculin null cells show higher endocytosis compared to WT cells but does not show increase in uptake on deadhering unlike WT cells. (n = WT Spread (234), WT Deadh (341), Vin-/- Spread (204), Vin-/- Deadh (369)). **(e)** The cells were pulsed with TMR-Dex in isotonic medium (Iso) or following a hypotonic shock (Hypo Iso) either in spread cells (control) or in suspension (Susp). Left panel shows representative images and box plot (right panel) shows normalized fluid uptake in spread cells and suspension. (n = Spread- Iso (352), Hypo-Iso (351); Suspension- Iso (236), Hypo-Iso (354)). **(f)** CHO cells expressing GFP- GPI was detached and allowed to settle on a glass bottom dish and imaged live. Cells are shifted to hypotonic medium for a minute and reverted to isotonic medium forming membrane protrusions indicative of a passive tension response as observed earlier (refer Supplementary Movie 3). Box plot shows median, 25th and 75th percentile, and whiskers show the standard deviation. Individual data points are overlaid on box plot where each data point is the mean intensity per cell. 'n' indicates total number of cells in each condition pooled from two different experiments with duplicates per experiment. *: $P < 0.001$, ns: not significant by Mann-Whitney U test. Scale bar, 10 μm.

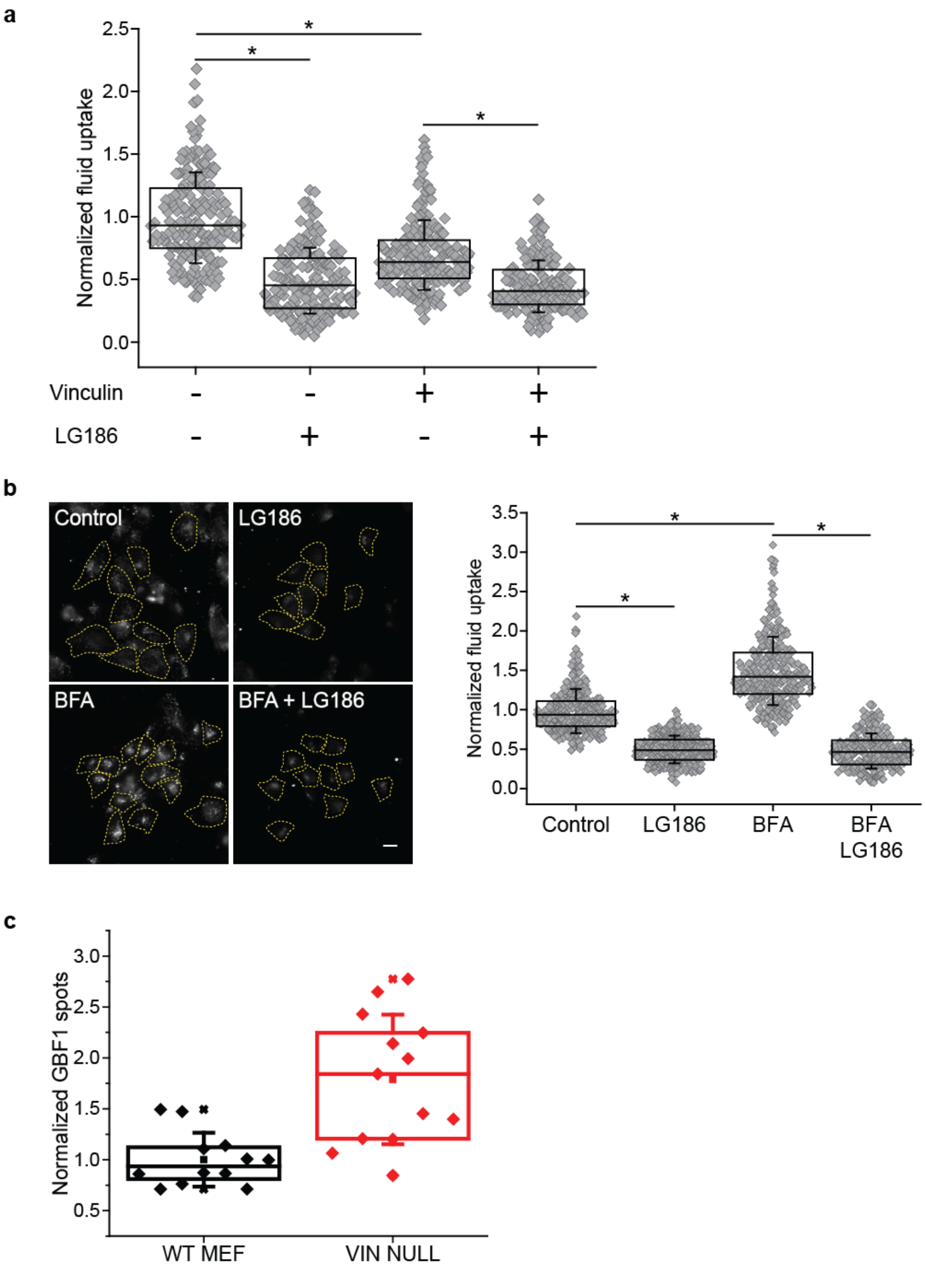

Supplementary Figure 11: (a) Vinculin null cells rescued by transfection with WT-Vinculin full length (+) and vinculin null cells (-) were treated with LG186 to check sensitivity to GBF1 inhibition of fluid-phase endocytosis. Cells treated with LG186 for 30 minutes were pulsed for 5 minutes with F-Dex, washed, fixed and imaged. Endocytic uptake per cell is quantified and normalized to the untreated Vin null case (Control). Note that adding back vinculin reduces the fluid-phase uptake. However, in both the vinculin null as well as in the cells in which vinculin is added back, endocytic uptake of the fluid-phase remains sensitive to LG186. (n = Vin-/- -Control (192), LG186 (184); VinWT -Control (196), LG186 (170)). **(b)** Vinculin cells were either untreated (Control) or treated with either LG186, BFA or BFA followed by LG186 treatment (BFA LG186). Cells were pulsed with F-Dex for 3 minutes and uptake per cell quantified as detailed in Methods, normalized to control, and plotted in the box plot (left). The box plot and images (right) show that BFA treatment causes an increase in fluid-phase uptake in vinculin null cells and treating with LG186 inhibits fluid-phase uptake even in BFA treated cells. This indicates that the CG pathway is capable of being further activated in the vinculin null cells and that the CG pathway that is operational and

responsive in the vinculin null cells. Data represent the mean intensity per cell from two different experiments with duplicates per experiment. (n = Control (309), LG186 (319), BFA (290), BFA+LG186 (247)). Box plot shows median, 25th and 75th percentile and whiskers show the standard deviation. Individual data points are overlaid on box plot where each data point is the mean intensity per cell. 'n' indicates total number of cells in each condition pooled from two different experiments with duplicates per experiment. *: $P < 0.001$, ns: not significant by Mann-Whitney U test. Scale bar, 10 μm. **(c)** GBF1 punctae in vinculin null cells are higher. GBF1-GFP is transfected in either WT-MEF or vinculin null MEF (Vin Null) and imaged in TIRF. The number of punctae per cell were counted and normalized to the surface area. The plot shows the average number of spots in each cell type normalized to those obtained in WT-MEF. Each point in the box plot represents measurements collected from a single cell (Number of cells: WT MEF= 12; VIN NULL= 13).

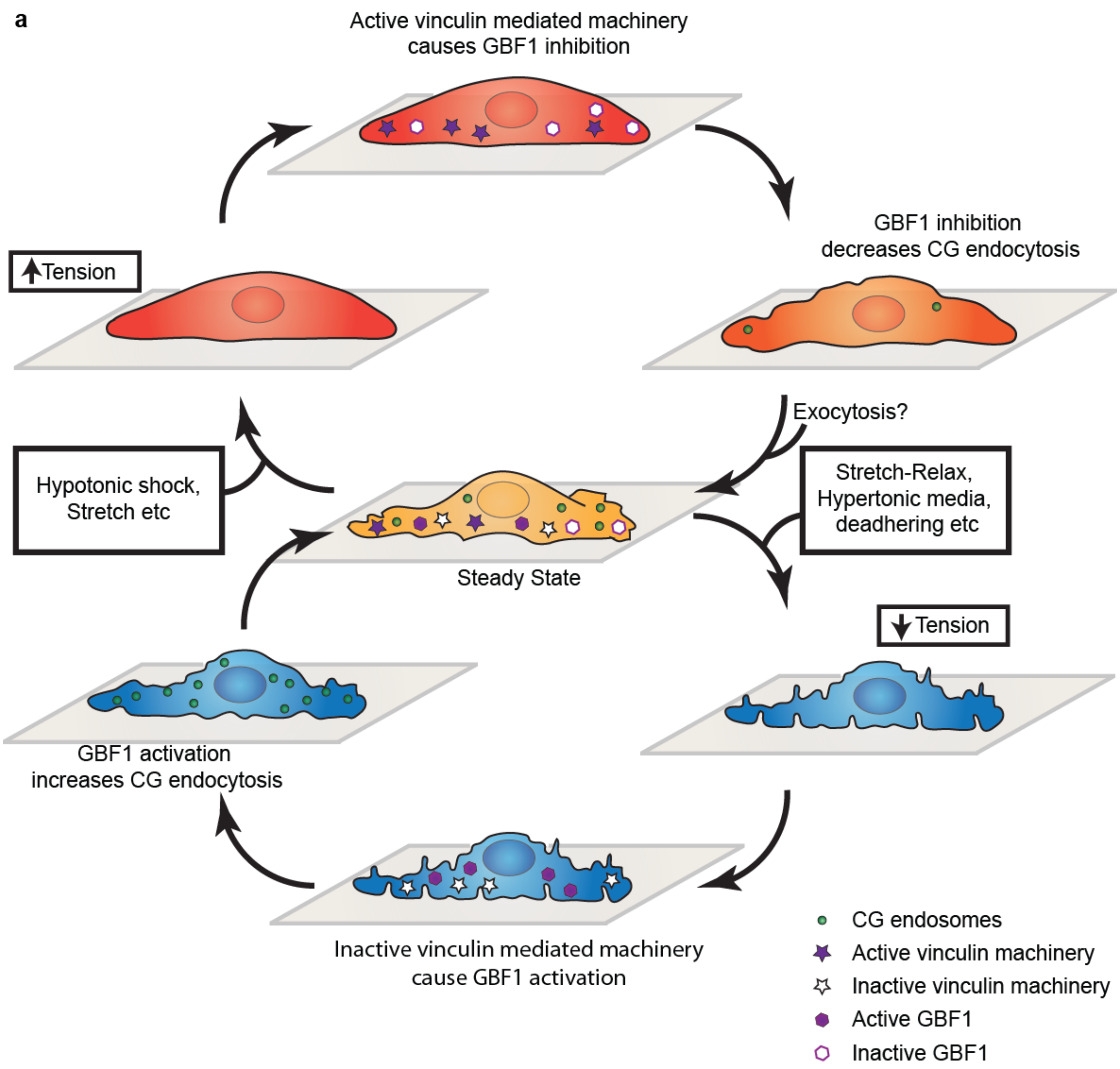

Supplementary Figure 12: CG pathway and membrane tension in maintaining membrane homeostasis. (a) Reduction of tension from its steady state leads to a passive response by the formation of reservoirs or VLDs. The decrease in the effective tension inactivates a vinculin-dependent machinery, resulting in an increase in active GBF1, which increases the CG pathway and rapid internalization of the excess membrane. This is a fast transient response that appears to restore the steady state. On the other hand, increasing the membrane tension from steady state activates vinculin dependent machinery, inhibiting the CG pathway, via the reduction of GBF1 recruitment. The increase in effective tension could also activate exocytic machinery which adds membrane resulting in restoration of the steady state. Thus, a vinculin dependent mechanochemical-regulation of the CG pathway through a negative feedback inhibition helps in maintaining plasma membrane tension homeostasis.

SUPPLEMENTARY MATERIALS AND METHODS

Cell lines: CHO (Chinese Hamster Ovary) cells stably expressing FR-GPI and human transferrin receptor TfR (IA2.2 cells)[8], HeLa[9], MEF (Mouse embryonic fibroblasts) cells[10], Caveolin null MEF[10], conditional null dynamin triple knockout MEF[11], vinculin null MEF[12], FR-AGS (Human adenogastric carcinoma cells stably transfected with FR-GPI)[13,14], IRSp53 null MEFs[14] and IRSp53 null MEFs stably expressing WT-IRSp53[14] were used for the assays. HF12 media (HiMEDIA, Mumbai, India) and DMEM (Invitrogen) supplemented with $NaHCO_3$ and L-Glutamine/Penicillin/Streptomycin solution (Sigma Aldrich) were used for growing CHO/FR-AGS cells and the different MEF lines respectively.

Constructs: pEYFP-mem construct was from Clontech[15], GFP-GBF1[7] was a kind gift from E. Sztul (U. Alabama, Birmingham), SecGFP-GPI[14] was made by site-directed mutagenesis, F64L and S65T in ecliptic-GPI received from Gero Miesenböck (University of Oxford), CLC-pmCherryC1 from Addgene (plasmid # 27680), AP2 shRNA and pSUPER empty vector[16] were a kind gift by Philippe Benaroch (Institut Curie, Paris) and vinculin constructs (Vin-WT, Vin A50I, Vin-CA,Vin-A50I-CA)[4] were a kind gift by Clare M. Waterman (NIH, USA).

Antibodies: anti-hTfR monoclonal antibody was purified from mouse hybridoma, OKT9[13] (National Centre for Cell Science, India, used at 10μg/ml) and anti–GM130 (Clone 35) from BD transduction laboratories (used at 1:100).

Synthesis of LG186: LG186 was synthesized freshly prior to experiments as reported previously[17] with slight modification as described below and used at 10μM dissolved in DMSO.

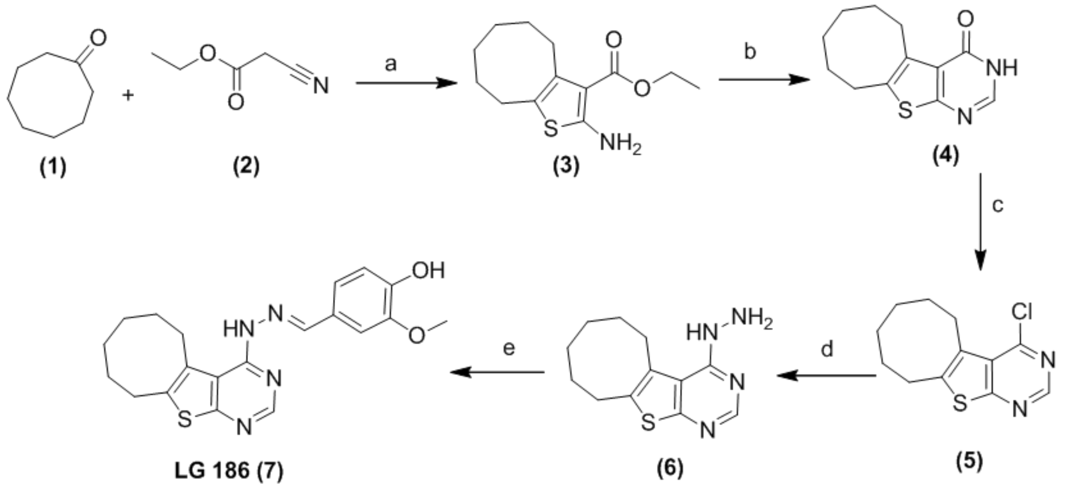

Reagent and conditions: Reagents and compounds obtained are mentioned as numbers and the conditions for the reaction are labelled alphabetically along with arrows in figure. a) S_8, morpholine, ethanol reflux 6 h; b) $HCONH_2$, 150°C, 5 h; c) $POCl_3$, DMF, rt. d) Hydrazine hydrate, methanol, rt. 2 h; e) Vanillin, rt. 2 h.

Compound 3: To a solution of cyclooctanone (**1**) (10 mmol) in ethanol (10 mL) were added sulfur (10 mmol), ethyl cyanoacetate (**2**) (10 mmol) and morpholine (4 mmol). The reaction

mixture was stirred at 60°C for 5 h. Upon completion of reaction (checked by TLC), evaporate the solvent and extracted with ethyl acetate and purified by column chromatography using dichloromethane. ^1H NMR (400 MHz, $CDCl_3$) δ: 1.28 (m, 5H), 1.39 (m, 2H), 1.50 (m, 2H), 1.56 (m, 2H), 2.54 (m, 2H), 2.75 (m, 2H), 4.21 (q, 2H), 5.86 (brs, 2H). LC-MS: 254 $(M+H)^+$.

Compound 4: Compound **3** was heated at 150°C in 5 mL formamide for 5 h. Upon cooling overnight, the product crystallized as slightly brownish crystals. The resulting crystals were collected and washed with a mixture of cold ethanol/water (1/1) to give the corresponding product in quantitative yield. ^1H NMR (400MHz, DMSO) δ: 1.27(m, 2H), 1.42 (m, 2H), 1.62(m, 4H), 2.87 (m, 2H), 3.06 (m, 2H), 8.5 (s, 1H), 11.4 (brs, 1H). LC-MS: 234.9 $(M+H)^+$.

Compound 5: Compound **4** was dissolved in hot DMF and then ice-cooled prior to the addition of $POCl_3$ (2 equivalents). Upon stirring overnight, the product precipitated out as white solid **5**, which was collected and washed with cold water and used for next step without purification.

Compound 7: Compound **5** was dissolved in methanol and then added 10 equivalent of hydrazine monohydrate. The mixture was stirred for 2 h and water was added. The resulting precipitate was filtered off and washed with cold water to obtain compound **6**, which was then treated with 1.2 equivalent of vanillin. The mixture was stirred for 2 h, diluted with water and extracted with dichloromethane. The organic layer was dried with MgSO4, filtered off and concentrated in vacuo and purified by column chromatography using ethyl acetate: hexane (4:6). ^1H NMR (400MHz, DMSO) δ: 1.27 (m, 2H), 1.46 (m, 2H), 1.62 (m, 2H), 1.68 (m, 2H), 2.85 (m, 2H), 3.19 (m, 2H), 3.88 (s, 3H), 6.84 (d, 1H), 7.60 (d, 1H), 7.79 (s, 1H), 8.30 (s, 1H), 9.45 (brs, 1H) 11.70 (brs, 1H). ^{13}C NMR: ^{13}C NMR (100 MHz, DMSO) δ 149.29, 148.97, 148.47, 146.76, 146.41, 141.11, 135.01, 132.59, 122.39, 118.77, 117.05, 116.46, 113.05, 58.20, 56.24, 26.95, 25.84, 25.72, 25.50. LC-MS: 383 $(M+H)^+$.

REFERENCES

1. Johnson, R. P. & Craig, S. W. An intramolecular association between the head and tail domains of vinculin modulates talin binding. *J. Biol. Chem.* **269,** 12611–12619 (1994).
2. Johnson, R. P. & Craig, S. W. F-actin binding site masked by the intramolecular association of vinculin head and tail domains. *Nature* **373,** 261–264 (1995).
3. Grashoff, C. *et al.* Measuring mechanical tension across vinculin reveals regulation of focal adhesion dynamics. *Nature* **466,** 263–6 (2010).
4. Case, L. B. *et al.* Molecular mechanism of vinculin activation and nanoscale spatial organization in focal adhesions. *Nat. Cell Biol.* **17,** 880–892 (2015).
5. Rothenberg, K. E., Scott, D. W., Christoforou, N. & Hoffman, B. D. Vinculin Force-Sensitive Dynamics at Focal Adhesions Enable Effective Directed Cell Migration. *Biophys. J.* **114,** 1680–1694 (2018).
6. Sun, L., Noel, J. K., Levine, H. & Onuchic, J. N. Molecular Simulations Suggest a Force-Dependent Mechanism of Vinculin Activation. *Biophys. J.* **113,** 1697–1710 (2017).
7. Gupta, G. D. *et al.* Analysis of endocytic pathways in Drosophila cells reveals a

conserved role for GBF1 in internalization via GEECs. *PLoS One* **4,** e6768 (2009).

8. Kumari, S. & Mayor, S. ARF1 is directly involved in dynamin-independent endocytosis. *Nat. Cell Biol.* **10,** 30–41 (2008).

9. Kalia, M. *et al.* Arf6-independent GPI-anchored protein-enriched early endosomal compartments fuse with sorting endosomes via a Rab5/phosphatidylinositol-3'-kinase-dependent machinery. *Mol. Biol. Cell* **17,** 3689–704 (2006).

10. Sinha, B. *et al.* Cells respond to mechanical stress by rapid disassembly of caveolae. *Cell* **144,** 402–13 (2011).

11. Park, R. J. *et al.* Dynamin triple knockout cells reveal off target effects of commonly used dynamin inhibitors. *J. Cell Sci.* **126,** 5305–12 (2013).

12. Janoštiak, R. *et al.* CAS directly interacts with vinculin to control mechanosensing and focal adhesion dynamics. *Cell. Mol. Life Sci.* **71,** 727–44 (2014).

13. Gupta, G. D. *et al.* Population distribution analyses reveal a hierarchy of molecular players underlying parallel endocytic pathways. *PLoS One* **9,** (2014).

14. Sathe, M. *et al.* Small GTPases and BAR domain proteins regulate branched actin polymerisation for clathrin and dynamin-independent endocytosis. *Nat. Commun.* **9,** 1835 (2018).

15. Kosmalska, A. J. *et al.* Physical principles of membrane remodelling during cell mechanoadaptation. *Nat. Commun.* **6,** 7292 (2015).

16. Dugast, M., Toussaint, H., Dousset, C. & Benaroch, P. AP2 clathrin adaptor complex, but not AP1, controls the access of the major histocompatibility complex (MHC) class II to endosomes. *J. Biol. Chem.* **280,** 19656–19664 (2005).

17. Boal, F. *et al.* LG186: An inhibitor of GBF1 function that causes Golgi disassembly in human and canine cells. *Traffic* **11,** 1537–51 (2010).
