# Supplementary material for: Mechanochemical feedback control of dynamin independent endocytosis modulates membrane tension in adherent cells
Source: Nat Commun. 2018 Oct 11;9:4217. doi: 10.1038/s41467-018-06738-5 (PMC6181995; doi:10.1038/s41467-018-06738-5)
Supplement: Supplementary file 3 — Description of Additional Supplementary Files [file 41467_2018_6738_MOESM3_ESM.pdf]

Description of Additional Supplementary Files

Supplementary Movie 1: Movie shows z- stack of confocal images of Iso fluid endosomes of the cells shown in Supplementary Fig. 3d. The z-stack movie starts from basal side of the cell to the top showing distribution of endosomes in each case.

Supplementary Movie 2: Movie shows z- stack of confocal images of Hypo-Iso fluid endosomes of the cells shown in Supplementary Fig. 3d. The z-stack movie starts from basal side of the cell to the top showing distribution of endosomes in each case.

Supplementary Movie 3: Movie shows time lapse of cell from Supplementary Fig. 10f showing membrane fluctuations during osmotic perturbations.
